# Determination of the molecular reach of the protein tyrosine phosphatase SHP-1

**DOI:** 10.1016/j.bpj.2021.03.019

**Published:** 2021-03-27

**Authors:** Lara Clemens, Mikhail Kutuzov, Kristina Viktoria Bayer, Jesse Goyette, Jun Allard, Omer Dushek

**Affiliations:** 1 Center for Complex Biological Systems, University of California Irvine, Irvine, California; 2 Sir William Dunn School of Pathology, University of Oxford, Oxford, United Kingdom; 3 EMBL Australia Node in Single Molecule Science, School of Medical Sciences University of New South Wales, Sydney, Australia; 4 ARC Centre of Excellence in Advanced Molecular Imaging, University of New South Wales, Sydney, Australia

## Abstract

Immune receptors signal by recruiting (or tethering) enzymes to their cytoplasmic tails to catalyze reactions on substrates within reach. This is the case for the phosphatase SHP-1, which, upon tethering to inhibitory receptors, dephosphorylates diverse substrates to control T cell activation. Precisely how tethering regulates SHP-1 activity is incompletely understood. Here, we measure binding, catalysis, and molecular reach for tethered SHP-1 reactions. We determine the molecular reach of SHP-1 to be 13.0 nm, which is longer than the estimate from the allosterically active structure (5.3 nm), suggesting that SHP-1 can achieve a longer reach by exploring multiple active conformations. Using modeling, we show that when uniformly distributed, receptor-SHP-1 complexes can only reach 15% of substrates, but this increases to 90% when they are coclustered. When within reach, we show that membrane recruitment increases the activity of SHP-1 by a 1000-fold increase in local concentration. The work highlights how molecular reach regulates the activity of membrane-recruited SHP-1 with insights applicable to other membrane-tethered reactions.

## Significance

Immune receptors transduce signals by recruiting (or tethering) cytoplasmic enzymes to their tails at the membrane. When tethered, these enzymes catalyze reactions on other substrates to propagate signaling. Precisely how membrane tethering regulates enzyme activity is incompletely understood. Unlike other tethered reactions, in which the enzyme tethers to the substrate, the substrate in this case is a different receptor tail. Therefore, the ability of the receptor-tethered enzyme to reach a substrate can be critical in controlling reaction rates. In this work, we determine the molecular reach for the enzyme SHP-1 and use it to quantify the impact of molecular reach on receptor signaling.

## Introduction

**Figure 1:**
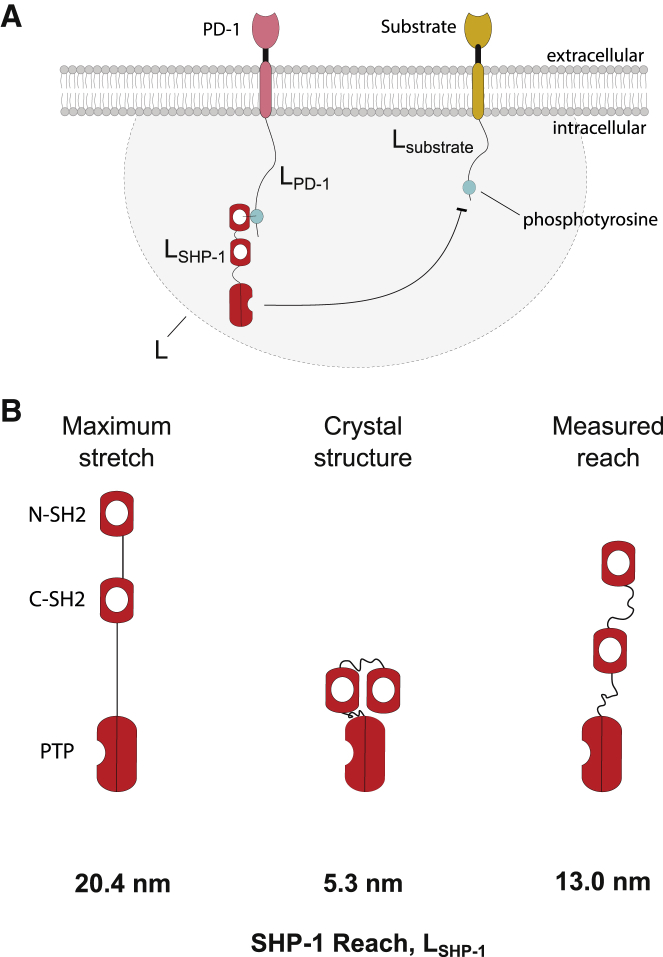
Molecular reach in immune receptor signal transduction. (*A*) Schematic of a tethered dephosphorylation reaction mediated by the tyrosine phosphatase SHP-1 (*red*) recruited to an inhibitory receptor (*pink*), such as PD-1, acting to dephosphorylate a membrane substrate (*orange*). The molecular reach of the reaction, *L* (*gray area*), is determined by the molecular reach of PD-1 (*L*_PD-1_), SHP-1 (*L*_SHP-1_), and the substrate (*L*_substrate_). It determines whether the substrate is within reach (within *gray area*) and the local concentration of SHP-1 when this is the case. (*B*) Estimates of SHP-1 molecular reach (*L*_SHP-1_) based on sequence (maximal stretch), crystal structure, and experimental measurement in this work. To see this figure in color, go online.

Immune receptor signal transduction proceeds by the recruitment of cytoplasmic enzymes to their unstructured cytoplasmic tails before they catalyze reactions on other membrane substrates (1, 2, 3). Well-studied examples include inhibitory checkpoint receptors, such as programmed cell death protein 1 (PD-1) on T cells, that can contain immunoreceptor tyrosine-based inhibition (ITIM) and switch (ITSM) motifs (2). Ligand binding induces phosphorylation of these motifs, which can then recruit the tyrosine phosphatases SHP-1 and SHP-2 by their SH2 domains. When tethered to inhibitory receptors, these promiscuous phosphatases are thought to dephosphorylate diverse membrane substrates, including the T cell receptor, the costimulation receptor CD28, the membrane adaptor LAT, and even autoinhibition of inhibitory receptors in *trans* (Fig. 1 *A*; (4, 5, 6, 7, 8)). Precisely how membrane recruitment regulates and directs the activity of SHP-1 is incompletely understood.

Biochemical and structural studies have clearly demonstrated that engagement of the SH2 domains of SHP-1 and its family member SHP-2 can induce a conformational change from a closed low-activity state into an open high-activity state (9, 10, 11, 12, 13, 14, 15, 16, 17, 18). When quantified, this binding-induced allosteric activation can increase catalytic rates by ∼80-fold (9), and therefore, this is a mechanism by which membrane recruitment can regulate enzyme activity and has motivated the development of therapeutic allosteric inhibitors (15).

Membrane recruitment can also tether SHP-1 in a small volume, increasing the local concentration of SHP-1 experienced by substrates (3). Tethering is prevalent in cellular signaling (19), and experimental and mathematical work has shown that it can dramatically increase local concentrations and hence reaction rates (16,20,21); it can also override enzyme specificity (22). How tethering impacts the local concentration of SHP-1 is presently unknown.

In contrast to previously studied tethered reactions (20,21,23), in which the enzyme tethers directly to the substrate, the situation is more complicated for immune receptors because the enzyme tethers to a receptor but acts in *trans* on a different membrane substrate (Fig. 1 *A*). Therefore, the potentially high local concentration that results from membrane recruitment may only be experienced by the small subset of substrates within reach. The molecular reach of the reaction (*L* in Fig. 1 *A*) (24) is a biophysical parameter that determines both the fraction of substrate within reach and, when this is the case, the local concentration (approximately *σ*^∗^ = 1/*L*^3^). Therefore, quantifying molecular reach is critical for understanding the impact of SHP-1 membrane recruitment.

The molecular reach of these reactions is presently unknown. There are three molecules that contribute to the reach: the receptor tail, the enzyme, and the substrate (24). Polymer models, such as the worm-like chain, have been used to estimate the reach of unstructured polypeptide chains based on the contour (*l*_*c*_) and persistence (*l*_*p*_) lengths as *L*_peptide_ = (*l*_*c*_*l*_*p*_)^1/2^ (20). This theoretical approach predicts a reach of *L*_PD-1_ = 3.0 nm for the ITSM of PD-1 located 55 amino acids (aa) from the membrane (using *l*_*c*_ = 55 × 0.4 nm, where 0.4 nm is the contribution of each aa and *l*_*p*_ = 0.4 nm for random aa sequences (20,25)). In the absence of other contributions, this reach is comparable to the lateral dimensions of receptor extracellular domains, implying that surface receptors must come into (or nearly into) contact to enable reactions.

However, SHP-1 may significantly contribute to this reach. SHP-1 tethers with its dominant N-terminal SH2 domain and catalyzes reactions with a C-terminus protein tyrosine phosphatase (PTP) domain (9,11,16) as shown in Fig. 1 *A*. Based on the structure of the allosteric open conformation of SHP-1 (14), the reach between the N-SH2 and the catalytic pocket is estimated to be 5.3 nm. However, SHP-1 may dynamically explore conformations not observed in crystals to achieve a longer reach. A potential upper bound can be estimated by assuming that all linkers are maximally stretched obtaining a reach of 20.4 nm (Fig. 1 *B*). However, the combination of structured domains, flexible linkers, and specific interactions between them makes it difficult to accurately predict the reach of multidomain proteins like SHP-1.

Here, we use surface plasmon resonance (SPR) to measure binding, catalysis, and molecular reach for tethered, autoinhibition *trans* reactions involving PD-1 and SHP-1 at 37°C. We find a reach of 13.0 nm for SHP-1, suggesting it dynamically explores a range of open conformations. The molecular reach shows that membrane recruitment can increase the activity of SHP-1 by a 1000-fold increase in local concentration, which is larger than the activity increase by allostery, and that clustering is required for PD-1-SHP-1 complexes to reach substrates. The work highlights the role of molecular reach in regulating the activity of tethered SHP-1 reactions, providing insights widely applicable to immune receptors.

## Methods

### SHP-1 molecular reach estimates from structure

Using the structure of SHP-1 in the open conformation (Protein Data Bank, PDB: 3PS5) and sequence data from UniProt (P29350), we can estimate a range of reach values for SHP-1. Direct measurement from the PDB structure of the N-SH2 binding site to the catalytic site gives a reach estimate of 5.3 nm. For our maximal reach estimate, we subdivide SHP-1 into three structured domains (N-SH2, C-SH2, and PTP) and two linker domains. For the structured domains, distances were measured from the structure in PDB: 3PS5: between binding pocket and linker for N-SH2, between two linkers for C-SH2, and from the linker to the active site for PTP. We then count the number of residues in the two intervening disordered linker domains and compute contour length assuming these are fully extended. Adding these five numbers together (N-SH2, linker, C-SH2, linker, PTP) yields a value of 20.4 nm. All measurements of structured domains were calculated using the measurement tool in PyMol.

### Peptides and SHP-1

All phosphopeptides were custom synthesized by Peptide Protein Research and were N-terminally biotinylated. Peptide sequences, including peptides conjoined with polyethylene glycol (PEG), are listed in Table 1. Human SHP-1 with an N-terminal 6× His tag was produced in *Escherichia coli* BL21-CodonPlus (DE3)-RIPL strain (Agilent Technologies, Santa Clara, CA) and purified on Ni^2+^-NTA agarose (Invitrogen, Carlsbad, CA) (16). Aliquots were stored at −80°C. On the day of experiment, SHP-1 was further purified by size-exclusion chromatography on an AKTA fast protein liquid chromatography system equipped with a Superdex S200 10/300 GL column (both from GE Healthcare Life Sciences; Marlborough, MA) equilibrated with 10 mM HEPES (pH 7.4), 150 mM NaCl, 3 mM EDTA, and 0.05% Tween 20 supplemented with 1 mM dithiothreitol. SHP-1 concentration was determined from absorbance at 280 nm measured on a Nanodrop ND-2000 spectrophotometer (Thermo Fisher Scientific, Waltham, MA).

**Table 1:** Peptides used in this study

Name	Sequence
PEG28-PD-1	biotin-(PEG)_28_-SVPEQTEY^∗^ATIVFPSG
PEG12-PD-1	biotin-(PEG)_12_-SVPEQTEY^∗^ATIVFPSG
PEG6-PD-1	biotin-(PEG)_6_-SVPEQTEY^∗^ATIVFPSG
PEG3-PD-1	biotin-(PEG)_3_-SVPEQTEY^∗^ATIVFPSG
PEG0-PD-1	biotin-SVPEQTEY^∗^ATIVFPSG
PD-1	biotin-SRAARGTIGARRTGQPLKEDPS AVPVFSVDYGELDFQ WREKTP EPPVPSVPEQTEY^∗^ATIVFPSG
SLAM	biotin-QLRRRGKTNHYQTTVEKK SLTIYAQVQKPGPLQKKLD SFPA QDPCTTIYVAATEPVPESVQET NSITVY^∗^ASVTLPES

Phosphotyrosines are denoted as Y^∗^.

### SPR

Experiments were performed on a Biacore T200 instrument (GE Healthcare Life Sciences) at 37°C and a flow rate of 10 *μ*L/min. Running buffer was the same as for size-exclusion chromatography. Streptavidin was coupled to a CM5 sensor chip (Cytiva, Marlborough, MA) using an amino coupling kit to near saturation, typically 10,000–12,000 response units (RU). Biotinylated peptides were injected into the experimental flow cells (FCs) for different lengths of time to produce desired immobilization levels (typically 25–100 RU). Concentrations of immobilized peptides were determined from the RU values as described in (16). The molar ratio of peptide/streptavidin was kept below 0.25 to avoid generating streptavidin complexes with more than one peptide. Usually, FC1 and FC3 were used as references for FC2 and FC4, respectively. Excess streptavidin was blocked with biotin (Avidity Biosciences, La Jolla, CA). Before SHP-1 injection, the chip surface was conditioned with 10 injections of the running buffer, and SHP-1 was then injected over all FCs; the duration of injections was the same for conditioning and SHP-1 injection (45 s).

### Solution assay for allosteric activation of SHP-1

The reaction mixture contained (final concentrations) 80 mM HEPES (pH 7.4), 1 mM dithiothreitol, 60 *μ*M PEG0-PD-1 peptide, 5% dimethyl sulfoxide (DMSO, vehicle), 10 mM p-nitrophenyl phosphate, and 0.1 *μ*M SHP-1; the reaction was started by adding SHP-1. The reaction mixtures were incubated at 37°C. Aliquots were withdrawn at appropriate time points, and dephosphorylation was stopped by addition of an equal volume of freshly prepared 80 mM HEPES (pH 7.4), 20 mM iodoacetamide, 100 mM Na_3_VO_4_. Absorbance at 405 nm was measured on the Nanodrop ND-2000. In the control, the quenching solution was added before SHP-1, and the mixture was kept either on ice or at 37°C for the duration of the time course. The efficiency of quenching was confirmed by the absence of a difference in absorbance between samples kept on ice or at 37°C.

### MPDPDE model and parameter fitting

We have previously derived a multicenter particle density partial differential equation (MPDPDE) model that accurately captures the stochastic and spatial features of tethered reactions in SPR (16). The nondimensional MPDPDE system is as follows:

$$\partial n_{A}/\partial t=-\left({\text{p}}_{1}+{\text{p}}_{5}\right)n_{A}+{\text{p}}_{2}n_{B}-4\pi {\left(3/2\pi \right)}^{3/2}{\text{p}}_{3}n_{A}n_{B}\int_{0}^{\infty }dr^{\text{′}}\left[{\left(r^{\text{′}}\right)}^{2}e^{-\frac{3{\left(r^{\text{′}}\right)}^{2}}{2}}Y\left(r^{\text{′}}\right)\right],$$

$$\partial n_{B}/\partial t={\text{p}}_{1}n_{A}-{\text{p}}_{2}n_{B},$$

$$\partial Y/\partial t=\frac{{\text{p}}_{1}n_{A}}{n_{B}}\left(X_{A}-Y\right)+\frac{{\text{p}}_{2}n_{B}}{n_{A}}\left(X_{B}-Y\right)-{\left(\frac{3}{2\pi }\right)}^{3/2}{\text{p}}_{4}e^{-\frac{3r^{2}}{2}}Y,$$

$$-2\pi {\left(\frac{3}{2\pi }\right)}^{3/2}{\text{p}}_{3}\frac{n_{B}Y}{r}\left(\int_{0}^{\infty }dr^{\text{′}}\int_{\left|r-r^{\text{′}}\right|}^{\left|r+r^{\text{′}}\right|}dq\left[qr^{\text{′}}e^{-\frac{3{\left(r^{\text{′}}\right)}^{2}}{2}}Y\left(r^{\text{′}}\right)\left(X_{B}\left(q\right)-1\right)\right]\right),$$

$$\partial X_{A}/\partial t=\frac{2{\text{p}}_{2}n_{B}}{n_{A}}\left(Y-X_{A}\right),$$

$$-4\pi {\left(\frac{3}{2\pi }\right)}^{3/2}{\text{p}}_{3}\frac{n_{B}X_{A}}{r}\left(\int_{0}^{\infty }dr^{\text{′}}\int_{\left|r-r^{\text{′}}\right|}^{\left|r+r^{\text{′}}\right|}dq\left[qr^{\text{′}}e^{-\frac{3{\left(r^{\text{′}}\right)}^{2}}{2}}Y\left(r^{\text{′}}\right)\left(Y\left(q\right)-1\right)\right]\right),$$

and

$$\partial X_{B}/\partial t=\frac{2{\text{p}}_{1}n_{A}}{n_{B}}\left(Y-X_{B}\right),$$

with initial conditions *n*_*A*_(*t* = 0) = 1, *n*_*B*_(*t* = 0) = 0, *X*_*A*_(*t* = 0, *r*) = 1, *X*_*B*_(*t* = 0, *r*) = 1, and *Y*(*t* = 0, *r*) = 1. In these equations, *A* refers to the free phosphorylated peptide, and *B* refers to the SHP-1-bound phosphorylated peptide. Specifically, *n*_*A*_ and *n*_*B*_ represent the concentration of free phosphorylated peptide and SHP-1-bound phosphorylated peptide, respectively, and *X* and *Y* describe the auto- and pair correlations, respectively. The five fitting parameters (p_1_, p_2_, p_3_, p_4_, and p_5_) are related to the five biophysical and biochemical constants as follows: p_1_ = *k*_on_ [SHP-1], p_2_ = *k*_off_, p_3_ = *k*_cat_(tethered) × [Peptide], p_4_ = *k*_cat_(tethered)*σ*^∗^, and p_5_ = *k*_cat_(solution) × [SHP-1]. The complete derivation can be found in our previous work (16).

These data exhibited nonspecific binding of the enzyme to the surface that differed in magnitude between the control and experimental FCs and, as a result, produced a different baseline before and after the SHP-1 injection. We therefore modified the original model to include nonspecific binding with rate *n*_ns_ that changed linearly with time (see Fig. S2) between the start (*p*_start_) and end (*p*_end_) of the SHP-1 injection. Therefore, the equation that we fitted directly to our SPR traces, which report the amount of SHP-1 bound over time, was as follows:

$$\text{Z}=0\;\text{for}\;t<p_{\text{start}},$$

$$\text{Z}=n_{\text{ns}}\cdot t+n_{B}\left(t\right)\;\text{for}\;p_{\text{start}}\leq t\leq p_{\text{stop}},$$

and

$$\text{Z}=n_{B}\left(t\right)\;\text{for}\;t\geq p_{\text{stop}},$$

where we set [SHP-1] = 0 (and hence *p*_1_ = *p*_5_ = 0) at *p*_stop_ to simulate the dissociation phase when the injection of SHP-1 stops and the injection of buffer resumes.

To fit the SPR data to the extended MPDPDE model, we use a simulated annealing algorithm (26) with at least 10^5^ steps and a temperature function decreasing to 0 as (1 − ([step]/10^5^)^4^). For initial guess, we used

$$k_{\text{ on}}=0.1\;\mu {\text{M}}^{-1}s^{-1},$$

$$\sigma^{\text{∗}}=544.6\;\mu \text{M}\;\left(L=14.5\text{nm}\right),$$

$$k_{\text{cat}}\left(\text{tethered}\right)=0.01\;\mu {\text{M}}^{-1}s^{-1},$$

and

$$k_{\text{cat}}\left(\text{solution}\right)=0.002\;\mu {\text{M}}^{-1}s^{-1}.$$

**Figure 2:**
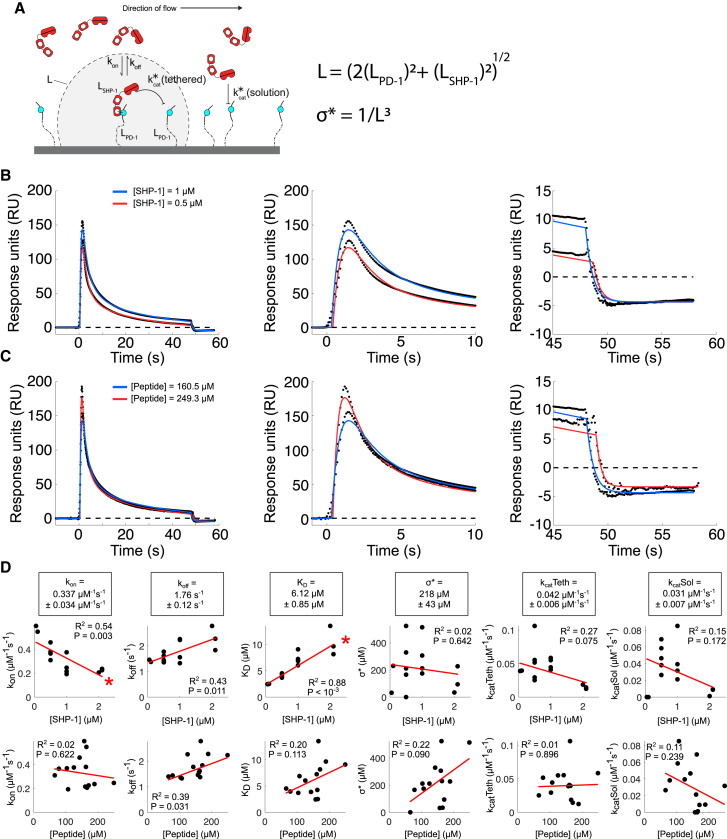
Extended SPR-based assay for tethered catalytic reactions can recover biophysical parameters independent of experimental conditions at 37°C. (*A*) Schematic of the SPR assay in which SHP-1 (analyte) is injected over immobilized phosphorylated PEG28-PD-1 peptides. (*B* and *C*) Representative SPR traces (*black dots*) and MPDPDE model fit (*solid lines*) for (*B*) two representative injected human SHP-1 concentrations and (*C*) two immobilized PEG28-PD-1 concentrations. Middle and right panels show early and late time data, respectively. (*D*) Fitted parameters (*black dots*) against SHP-1 concentration (*top row*) and PEG28-PD-1 concentrations (*bottom row*) with linear regression (*red line*; *R*^2^ and *p*-values without corrections). Red asterisks denote significant correlations at 5% level for Bonferroni-corrected *p*-values. Averages and SEMs of fitted parameters are shown in boxes above corresponding plots (n = 14). All parameters are summarized in Table 2. To see this figure in color, go online.

For the initial guess of *k*_off_, we first fit an exponential curve to the SPR time series data in the dissociation phase after SHP-1 injection ceases (e.g., after *t* = 45 s in Fig. 2 *A*). We find the parameters generated by simulated annealing are in close agreement with parameters found from MATLAB’s (The MathWorks Natick, MA) least-squares curve fitting (lsqcurvefit) function (data not shown). However, the sum of square error for the parameters found using simulated annealing is consistently smaller. We perform simulated annealing three times on each data set, using the fit with the lowest sum of square error for our analysis. All model evaluation and fitting are implemented in MATLAB 2017b.

To test for under-constrained parameter fitting, we perform a Markov chain Monte Carlo (26,27). Specifically, we use the Metropolis-Hastings algorithm (27,28) with flat, unbounded priors for *p*_1_, *p*_2_, *p*_3_, *p*_4_, *p*_5_, *n*_ns_, and *p*_*stop*_. We bounded *p*_start_ to be less than 0.3 s. The Metropolis algorithm proposes configurations using a perturbation size that is adaptive, increasing or decreasing until the acceptance rate is 0.44 (26). We repeat parameter proposals until the sequence of samples has reached a stationary distribution, which we define when the third quarter and fourth quarter of the sequence have the same distribution according to the Kolmogorov-Smirnov statistics. The resulting posteriors are shown in Fig. S3. All of the parameters exhibit compact posterior distributions in which most of the probability mass is concentrated in a single peak. Some weak correlations are evident, but these are away from the peaks. This suggests that the parameters can be independently determined.

### Estimation of molecular reach

The molecular reach of the reaction, *L*, in our SPR assays is influenced by the reach for the tether, *L*_tether_, and the reach of the enzyme, *L*_SHP-1_. For a worm-like chain model, the probability density of a site on the molecule at location $\vec{x}$ is

(1) $$P\left(\vec{x};L_{\text{X}}\right)={\left(\frac{3}{2\pi L^{2}}\right)}^{3/2}\text{exp}\left(-\frac{3\vec{x}\cdot \vec{x}}{2L^{2}}\right),$$

where *L*_X_ is a property of the molecule. For a worm-like chain model, *L*_X_ = $\sqrt{2l_{c}l_{p}}$, where *l*_*c*_ is the contour length and *l*_*p*_ is the persistence length, but we note that Eq. 1 arises in more general molecular models, so we use it to describe the behavior of the enzyme, without the interpretation of *L*_X_ in terms of a contour length and persistence length. In (16), we show that this leads to a local concentration kernel

(2) $$\sigma \left(r\right)={\left(\frac{3}{2\pi L^{2}}\right)}^{3/2}\text{exp}\left(-\frac{3r^{2}}{2L^{2}}\right),$$

where *r* is the distance between the anchors of the two tethers and

(3) $$\text{L}\;=\sqrt{L_{\text{tether}}^{2}+L_{\text{tether}}^{2}+L_{\text{SHP}-1}^{2}}$$

and

(4) $$=\sqrt{2\times L_{\text{tether}}^{2}+L_{\text{SHP}-1}^{2}}.$$

For disordered domains and PEG linkers, we interpret *L* in terms of the worm-like chain model (20,29), so the reach can be estimated from the contour length and the persistence length of the domain. For the constructed PEG-PD-1 peptides, the contour length (from the surface anchor to binding site of SHP-1) is the number of PEG linkers *N*_PEG_ times the length of a single PEG, *l*_PEG_ ∼0.4 nm (30). From this, we derive an approximation for the reach of SHP-1,

(5) $$L^{2}=4\times N_{\text{PEG}}\times l_{\text{PEG}}\times l_{p}+L_{\text{SHP}-1}^{2},$$

predicting that the reach is given by the intercept of the line *L*^2^ vs. *N*_PEG_.

### Uncertainty quantification for derived parameters

For each PEG length, *L*^2^ is calculated by averaging the fitted parameter *σ*^∗^ for all replicates and transforming the average to a single *L*^2^-value for the peptide. Error propagation is used to convert the standard deviation of *σ*^∗^ to an error for *L*^2^.

**Figure 3:**
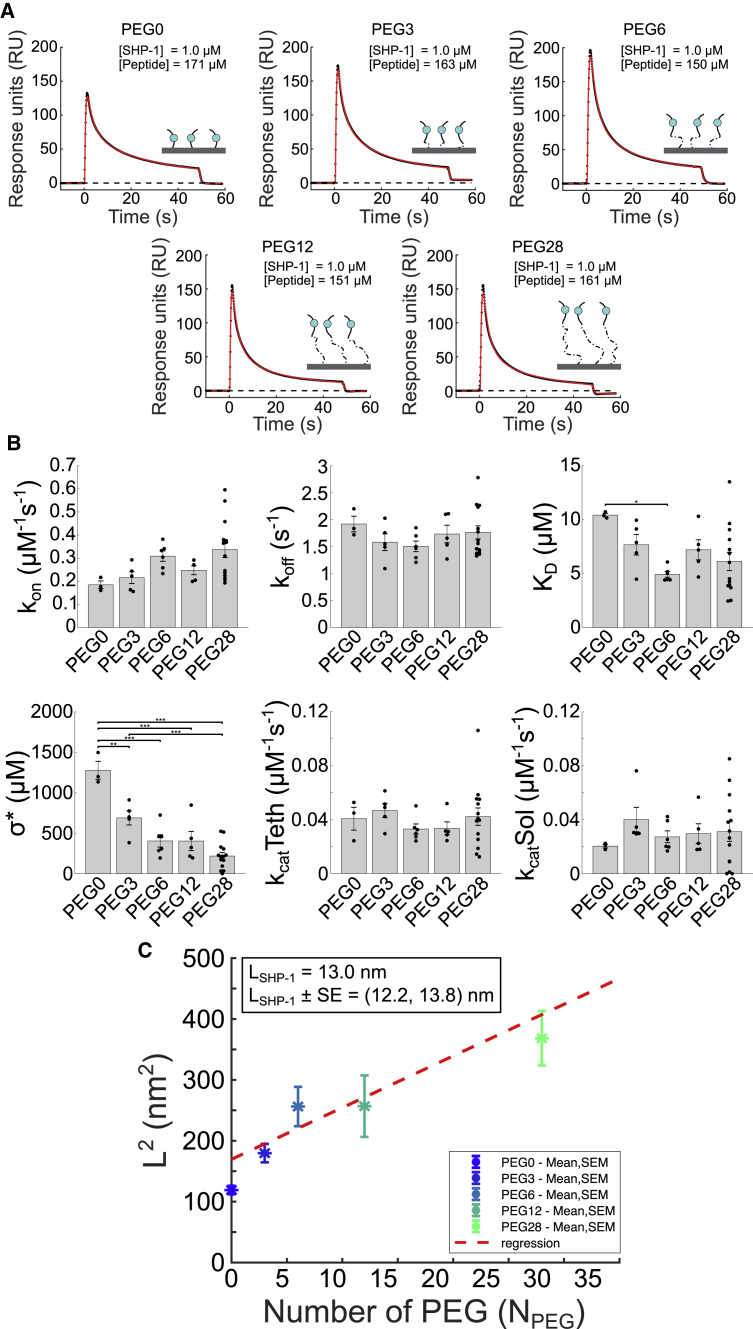
Isolating the molecular reach of SHP-1 by varying PEG-PD-1 tether lengths. (*A*) Representative SPR traces (*black dots*) and extended MPDPDE model fits (*red lines*) for the indicated number of PEG linkers (*N*_PEG_ = 0, 3, 6, 12, 28). (*B*) Averages and SEMs for fitted parameters at indicated PEG linker length. Individual data points are plotted as black dots. Pairwise multiple *t*-test of parameters and PEG lengths is shown for (^∗^) 0.05, (^∗∗^) 0.01, and (^∗∗∗^) 0.001 significance. Significant differences are largely observed for *σ*^∗^, which determines the molecular reach of the reaction (*L* = (*σ*^∗^)^−1/3^). All parameters are summarized in Table 2. (*C*) Average squared molecular reach of reaction plotted against number of PEG linkers. Red dashed line indicates regression (*p*-value = 10^−11^; see Methods). The indicated molecular reach of SHP-1 is estimated by the vertical intercept using the regression line. To see this figure in color, go online.

Best-fit lines with associated R-values and *p*-values for PEG28-PD1 parameters versus phosphatase and peptide concentrations, shown in Figs. 2 and 3, were determined using MATLAB’s robust fitlm function.

We use MATLAB’s anova1 and multcompare functions to conduct multiple comparison *t*-tests on paired PEG-peptide parameters to establish significant differences. Pairs that are significantly different at the 0.05, 0.01, and 0.001 level are shown.

### Implications of reach for reactions at the cell membrane

For a given receptor density *ρ*_0_, the effective concentration experienced by a substrate is

(6) $$C_{\text{eff}}=\iint \sigma \left(r\right)\rho_{0}dA$$

and

(7) $$={\left(\frac{3}{2\pi }\right)}^{3}\frac{\rho_{0}}{L_{\text{rxn}}}.$$

Under the assumption that the receptors are uniformly distributed on the cell, we estimate the surface density to be ∼2 × 10^−4^ nm^−2^. We obtain this by using copy numbers of typical inhibitory receptors like signaling lymphocyte activation molecule (SLAM) and PD-1 (20,000–80,000 molecules per cell (31)) and assuming the T cell is approximately a sphere of radius 5 *μ*m. For comparison, the cytosolic SHP-1 concentration is estimated to be 1 *μ*M (31).

Because tethered reactions are randomly distributed on the membrane, some substrate molecules are inaccessible to the enzyme. We determine what fraction of the substrate is accessible by a receptor-SHP-1 complex for a given reach of reaction and receptor density. To do this, we calculate the probability of at least one receptor-SHP-1 complex being within a circle of radius L of any given substrate. We assume finding a number of activated, SHP-1-bound receptor molecules within a disk around the substrate is Poisson distributed, with *λ* = *πρ*_0_$L_{\text{rxn}}^{2}$ and *ρ*_0_ estimated above. We use this to determine the probability of at least one receptor-SHP-1 complex within reach,

(8) $$P_{\geq 1}=1-\text{exp}\left(-\pi \rho_{0}L_{\text{rxn}}\right).$$

## Results

### Extended SPR assay determines biophysical parameters for tethered reactions by SHP-1

We used an SPR-based assay to determine the biophysical parameters for PD-1-tethered SHP-1 reactions (Fig. 2 *A*). We injected human SHP-1 over a surface coated with phosphorylated ITSM peptide from human PD-1 initially coupled to 28 repeats of PEG (PEG28-PD1), and SHP-1 binding (via its SH2 domains) was monitored by SPR over time (Fig. 2, *B* and *C*). Because SH2 domains only bind phosphorylated peptides, it was observed that although binding initially increases (between 0 and ∼2.5 s), it rapidly decreased as a result of PD-1-tethered SHP-1 dephosphorylating other PD-1 molecules within reach in *trans* (between 2.5 and ∼20 s). These tethered reactions were self-limiting because fewer phosphorylated PD-1 molecules remain within reach over time, and instead, dephosphorylation could only take place by SHP-1 acting directly from solution in *cis* (between ∼20 and ∼50 s). Intuitively, the molecular reach determines the fraction of the surface that can be dephosphorylated by tethered SHP-1 at a given initial density of phosphorylated peptides, with a larger fraction indicating a longer reach.

We previously reported an MPDPDE model that fits these multiphasic SPR traces and is able to recover binding, catalysis, and reach parameters (16). However, in our previous work we performed all experiments at 10°C to increase binding, decrease reaction rates, and improve instrument stability. Here, we performed experiments at 37°C and routinely found a difference in the baseline SPR signal between the start (*t* = 0 s) and the end (*t* ∼50 s) of the SHP-1 injection, for example, visible in Fig. 2, *B* and *C*. Using alkaline phosphatase, we found that this difference was not a result of phosphate mass being lost from the surface (Fig. S1) but rather by nonspecific binding of the enzyme (Fig. S2). We therefore extended the MPDPDE model to capture nonspecific binding by introducing three additional parameters (*p*_start_, *p*_stop_, *p*_*nsb*_), and in addition, we included the dissociation phase in the fit; see Methods for details.

With these changes, we found that the extended eight-parameter MPDPDE model (*k*_on_, *k*_off_, *k*_cat_(tethered), *σ*^∗^, *k*_cat_(solution), *p*_start_, *p*_stop_, *p*_*nsb*_) closely fits the 37°C SPR data (e.g., Fig. 2, *B* and *C*). We perform Markov chain Monte Carlo analysis to assess whether the parameters can be uniquely identified and found this to be the case (Fig. S3).

We next determined whether the fitted parameters were independent of the SHP-1 and PEG28-PD-1 concentrations. We repeated the experiments at various concentrations and found that the fitted parameters associated with catalysis (*k*_cat_(tethered), *k*_cat_(solution), and *σ*^∗^) were independent of concentrations (Fig. 2 *D*, correlations are not significant). However, binding parameters (*k*_on_, *k*_off_, and *K*_D_ = *k*_off_/*k*_on_) exhibited a correlation with SHP-1 concentration, with a significant correlation for *k*_on_ and *K*_D_ after correcting for multiple hypotheses (indicated by *red asterisks* in Fig. 2 *D*). This correlation may arise because higher concentrations of SHP-1 could lead to steric crowding effects on the surface, whereby volume exclusion reduces the ability for more SHP-1 molecules to bind to the surface reducing apparent binding. We concluded that the catalytic parameters, including reach, can be determined using this fitting procedure.

### Isolating the molecular reach of SHP-1 by varying the tether length

The molecular reach of the reaction, *L* = (*σ*^∗^)^−1/3^, involves two components: the reach of the PEG-peptide tether and the reach of the enzyme. As the reach contributed by the tether is progressively decreased (e.g., by shorter tethers), eventually the molecular reach of the reaction will be wholly determined by the reach of the enzyme. Indeed, assuming that the reach of the tethers and enzyme can be effectively modeled by worm-like chains, an equation can be derived to relate *L* with the contour length of the tether (Eq. 4; see Methods). This model predicts that the squared molecular reach of the reaction should be linearly related to the length of the tether (Eq. 5), with the reach of the enzyme being the vertical intercept (i.e., when the tether length is nil).

Therefore, we performed the SPR-based assay using a different number of PEG repeats (*N*_*PEG*_ = 0, 3, 6, 12, 28) coupled to the same short PD-1 ITSM peptide (Fig. 3 *A*). As before, the extended MPDPDE model was able to fit the data and produced binding and catalysis parameters that were similar for different length PEG linkers with the exception of *σ*^∗^, which progressively increased as the number of PEG linkers was reduced (Fig. 3 *B*). This is expected because with shorter PEG linkers, the local volume that SHP-1 is confined to decreases, thereby increasing local concentration.

As expected, the squared molecular reach of the reaction (determined by converting the averaged *σ*^∗^ to *L*) increased with the number of PEG linkers (Fig. 3 *C*). Using regression on all data except PEG0, we determined the vertical intercept, and hence the molecular reach of SHP-1, to be *L*_SHP-1_ = 13.0 ± 0.8 nm. This value is between estimates obtained using crystal structure and maximal stretch (Fig. 1 *B*).

When directly coupling the PD-1 peptide without any PEG repeats (PEG0), we found a molecular reach of 10.9 ± 0.3 nm. Although this value is also within theoretical estimates for the reach of SHP-1 and similar to the value obtained by the intercept method above, we reasoned that it may be less accurate because this very short peptide can introduce steric hindrance to binding and catalysis (e.g., by more readily adopting conformations in which the binding site is near the surface), which is reflected in the larger value of *K*_D_ and smaller value of *k*_cat_(solution) that this peptide produces compared with peptides with PEG linkers.

### PD-1 contributes less than SHP-1 to the molecular reach of the reaction

**Figure 4:**
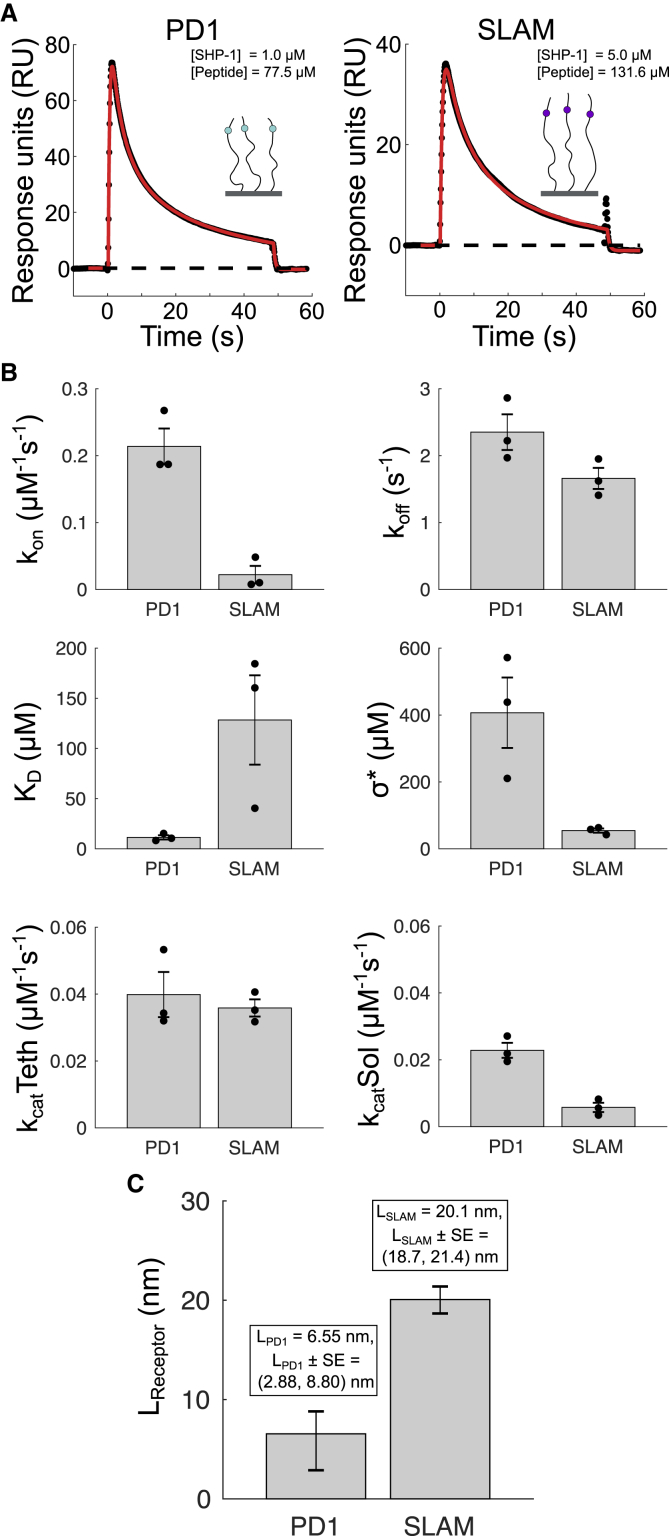
Contribution of PD-1 and SLAM cytoplasmic tails to the molecular reach of the reaction. (*A*) Representative SPR traces (*black dots*) and extended MPDPDE model fits (*red lines*) for the singly phosphorylated PD-1 (55 aa to phosphorylated tyrosine) and SLAM (69 aa to phosphorylated tyrosine) peptides. (*B*) Averages and SEMs for fitted parameters. Individual data points are plotted as black dots. PD-1 exhibits a larger local concentration (*σ*^∗^) consistent with a shorter molecular reach. All parameters are summarized in Table 2. (*C*) Average molecular reach (± SE) for PD-1 and SLAM calculated by parsing out the reach of SHP-1 using *L*_PD-1 or SLAM_ = ((*L*^2^ − $L_{\text{SHP}-1}^{2}$)/2)^1/2^, where *L* is the molecular reach of the reaction calculated from *σ*^∗^ in (*B*) and *L*_SHP-1_ = 13.0 nm. To see this figure in color, go online.

Given that the molecular reach of the reaction is determined by both the enzyme and tether, we next sought to determine the molecular reach of the receptor tail. We injected SHP-1 over immobilized peptide corresponding to the cytoplasmic tail of PD-1 from the membrane to the ITSM. This N-terminally biotinylated peptide contained 64 aa, with the phosphorylated tyrosine in the ITSM being 55 aa from the membrane (position 248 in the native sequence). The extended MPDPDE model was fitted to the SPR traces (Fig. 4 *A*) and provided estimates of the biophysical parameters (Fig. 4 *B*).

Using the value of *σ*^∗^, we calculated the combined molecular reach of the reaction for PD-1-bound SHP-1 acting on PD-1 to be 16 nm. Given that we already obtained an estimate for the reach of SHP-1, we were able to back calculate the reach of PD-1 (see Eq. 4) to be 6.55 nm (Fig. 4 *C*). Thus, we find that PD-1 contributes less to the overall molecular reach of the reactions compared with the SHP-1 reach contribution of 13.0 nm.

We note that the worm-like chain model would predict a 3.0 nm reach for the PD-1 peptide we have used, assuming a persistence length of 0.4 nm that applies to random aa chains (20,25). Therefore, the experimentally measured reach of PD-1 appears to be twice that predicted by the worm-like chain model, suggesting a preference for extended conformations of this peptide.

The binding affinity between SHP-1 and singly phosphorylated PD-1 was determined to be 11 ± 2 *μ*M. Using a different assay, Hui et al. (5) reported an affinity of 4.28 *μ*M. The ∼2-fold higher affinity they report is likely a result of using a doubly phosphorylated PD-1 peptide.

The ratio of *k*_cat_(tethered)/*k*_cat_(solution) provides an estimate for the strength of allosteric activation of SHP-1 upon SH2-domain binding to PD-1. We find a modest twofold increase in activity from this effect (Fig. 4 *B*). Because larger fold increases have been reported previously (9,11,16), we further explored this finding. First, we used a standard solution assay whereby SHP-1 acted on a low-molecular-weight synthetic substrate and confirmed that catalytic activity increased only twofold upon addition of a phosphorylated PD-1 peptide (Fig. S4). Second, we previously reported a larger allosteric activation for murine SHP-1 binding to the inhibitory receptor LAIR-1, but at 10°C, and therefore performed experiments at this lower temperature, finding again only a modest increase in activity (Fig. S5). We conclude that human SHP-1 exhibits only modest allosteric activation upon binding to singly phosphorylated PD-1.

As a positive control to ensure our SPR-based assay is sensitive to reach, we repeated the experiments using the longer cytoplasmic tail of SLAM, a surface receptor that is also known to recruit SHP-1 (32). The N-terminally biotinylated peptide contained 77 aa, with the phosphorylated tyrosine in the ITSM being 69 aa from the membrane (position 327 in the native sequence). Performing the analysis as for PD-1, we find that the molecular reach contributed by SLAM is 20 nm (Fig. 4). This is markedly more than the reach of SHP-1 and comprises 72% of the predicted contour length for the SLAM peptide (*l*_*c*_ ∼69 × 0.4 nm = 27.6 nm). This suggests that SLAM has a larger persistence length than would be expected for random aa's and/or is otherwise biased toward extended conformations.

Interestingly, we observed a larger 6.2-fold allosteric activation for SHP-1 interacting with SLAM (Fig. 4 *B*), and this is highlighted when plotting the ratio of *k*_cat_(tethered)/*k*_cat_(solution) across all experimental conditions (Fig. S6). However, this allosteric activation for SLAM was a result of a lower *k*_cat_(solution), not a higher *k*_cat_(tethered), compared with PD-1. We also observe a much smaller on rate for SHP-1 binding to SLAM compared with PD-1. A possible explanation for both observations is that the SLAM peptide may have fewer configurations in which the phosphotyrosine is available for interaction with SHP-1 when in solution.

Lastly, we noted that temperature had a large impact on these tethered reactions. We observed ∼2-fold slower binding kinetics, ∼10-fold slower catalytic rates, and an ∼4.5-fold larger value of *σ*^∗^ (960 vs. 210 *μ*M) at 10°C compared to 37°C using PEG28-PD-1 (Table 2). This underlined the importance of the extended SPR assay in overcoming the technical issues associated with making measurements at physiological temperatures.

**Table 2:** Average biophysical parameter values for each peptide

Substrate	N	*k*_on_ (*μ*M^−1^ s^−1^)	*k*_off_ (s^−1^)	*K*_D_ (*μ*M)	L (nm)	*σ*^∗^ (*μ*M)	*k*_cat_(tethered) (*μ*M^−1^ s^−1^)	*k*_cat_(solution) (*μ*M^−1^ s^−1^)
PEG0	3	0.19 ± 0.02	1.9 ± 0.1	10.4 ± 0.2	10.9 ± 0.3	1300 ± 100	0.041 ± 0.008	0.020 ± 0.001
PEG3	5	0.22 ± 0.03	1.6 ± 0.2	8.0 ± 1.0	13.4 ± 0.6	690 ± 90	0.047 ± 0.005	0.040 ± 0.009
PEG6	6	0.31 ± 0.02	1.5 ± 0.1	4.9 ± 0.3	16.0 ± 1.0	410 ± 77	0.033 ± 0.004	0.027 ± 0.004
PEG12	5	0.25 ± 0.02	1.7 ± 0.2	7.2 ± 0.9	16.0 ± 1.6	400 ± 100	0.034 ± 0.005	0.030 ± 0.007
PEG28	14	0.34 ± 0.03	1.8 ± 0.2	6.1 ± 0.8	19.7 ± 1.3	210 ± 40	0.042 ± 0.006	0.031 ± 0.007
PEG28 (10°C)	8	0.28 ± 0.03	0.8 ± 0.06	2.9 ± 0.2	12.0 ± 1.1	960 ± 260	0.0036 ± 0.0006	0.0047 ± 0.0007
PD1	3	0.21 ± 0.03	2.4 ± 0.3	11.0 ± 2.0	16.0 ± 1.4	400 ± 100	0.040 ± 0.007	0.023 ± 0.002
SLAM	3	0.02 ± 0.01	1.7 ± 0.2	130 ± 40	31.2 ± 1.2	55 ± 6	0.036 ± 0.003	0.0058 ± 0.001

All experiments conducted at temperature 37°C except where noted. Uncertainty is computed as the SE of the mean among the *N* different experiments (shown in second column).

### Control of surface receptor signaling by the molecular reach of SHP-1

**Figure 5:**
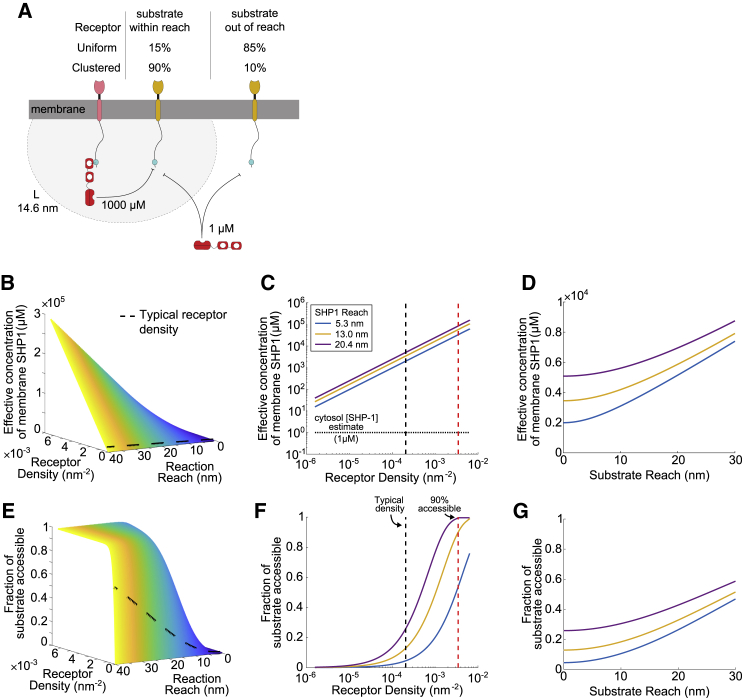
Tethering of SHP-1 to inhibitory receptor generates high membrane concentrations but poor coverage unless receptor coclusters with substrates. (*A*) Schematic of SHP-1 reactions with substrates, demonstrating tethered reaction (*left* SHP-1 molecule) and solution reaction (*right* SHP-1 molecule). (*B*–*D*) Effective concentration of receptor-SHP-1 complex experienced by a substrate and (*E*–*G*) fraction of substrate within reach by receptor-SHP-1 complexes under different conditions: (*B* and *E*) versus the molecular reach of reaction and the density of receptor-SHP-1 complex on the membrane. The cell surface receptor-SHP-1 density estimate is shown with black dashed line; (*C* and *F*) versus receptor-SHP-1 density for different estimates of SHP-1 molecular reach and fixed receptor and substrate molecular reach (6.55 and 0 nm, respectively). The density of receptor-SHP-1 complexes based on a uniform distribution is shown with a black dashed line in (*B*), (*C*), (*E*), and (*F*). Density required to reach 90% of substrate (0.0034 nm^−2^) is shown with red dotted line in (*F*). Estimate of cytosol SHP-1 concentration (1 *μ*M) is shown with dotted horizontal line in (*C*); and (*D* and *G*) versus substrate reach (for fixed reach of receptor and indicated reach of SHP-1) and receptor-SHP-1 uniform density (∼2 × 10^−4^ nm^−2^). Colored lines in (*C*), (*D*), (*F*), and (*G*) refer to the theoretical and experimental estimates of the molecular reach of SHP-1 (Fig. 1*B*), see legend in (*C*). To see this figure in color, go online.

We next used a mathematical model to explore how molecular reach regulates the activity of SHP-1 upon recruitment to an inhibitory receptor confined to the two-dimensional membrane (Fig. 5 *A*). The difference in receptor distribution between our experiments and the membrane is that in our experiments, the receptor is randomly distributed in three dimensions. By using a mathematical model in the previous section that accounted for this three-dimensional geometry, we are able to produce geometry-independent parameters that can now be used to predict the impact of reach for any receptor distribution, including the two-dimensional membrane distribution. Using PD-1 as a prototype, we calculated the combined reach of receptor-SHP-1 complexes as 14.6 nm $\left(\sqrt{L_{\text{PD}-1}^{2}+L_{\text{SHP}-1}^{2}}\right)$. Using this number, we first consider the effective concentration of SHP-1 that a substrate would experience when receptors are randomly distributed on the membrane. At typical physiological densities of inhibitory receptor, this effective concentration is ∼1000 *μ*M (Fig. 5, *B* and *C*), which is ∼1000-fold larger than the ∼1 *μ*M concentration of SHP-1 in the cytosol, assuming it is uniformly distributed (16,31).

In these tethered reactions, even though the effective concentration can be large, the coverage can in principle be low because a random or uniform distribution of surface receptors can allow some substrates to be out of reach (Fig. 5 *A*). We therefore calculated the fraction of substrates that can be accessed by receptor-SHP-1 complexes for different values of the molecular reach of the reaction and receptor density (Fig. 5, *E* and *F*). If receptors are uniformly distributed on the cell surface, we estimate that they are only able to achieve a low ∼15% coverage of the substrates (Fig. 5, *E* and *F*). Coclustering of receptors and substrates would lead to a higher local density and could therefore be important to improve both the effective concentration and coverage. We find that a clustered density of 0.0034 nm^−2^ (∼10-fold higher than a uniform estimate) is required to achieve a 90% coverage.

We next explored the contribution of the substrate reach to both concentration and coverage. Previously, we noted that the number of aa's between the membrane and activating or inhibiting tyrosine motifs differed with a median of 33 aa (or 13 nm) or 65 aa (or 26 nm), respectively (16). We therefore repeated the calculations by increasing the contribution of the substrate reach from 0 nm (used in Fig. 5, *B*, *C*, *E*, and *F*) to a maximum of 30 nm and found a gradual increase in effective concentration (Fig. 5 *D*) and coverage (Fig. 5 *G*). Within this realistic range of substrate reaches and with uniform distributions, it was not possible to achieve high coverage (e.g., 90%), underlining the importance of receptor-substrate coclustering. We note that including diffusion in the model is unlikely to change these conclusions if the rate of substrate phosphorylation is high because although receptor-SHP-1 complexes may diffuse to reach a substrate, a low coverage would mean that the same fraction of substrates would be out of reach and actively signaling at any given time.

## Discussion

We provide the first estimates of the molecular reach for an enzyme at physiological temperatures. The molecular reach has several implications for how membrane recruitment regulates and directs the activity of SHP-1.

The two-state allosteric activation model of SHP-1 (33,34) is based on crystal structures showing a closed autoinhibitory conformation, in which the N-SH2 domain blocks the catalytic pocket (13), and an open conformation, in which the N-SH2 is rotated, exposing the catalytic pocket (14). Interestingly, the molecular reach of SHP-1 that we report when tethered in the higher activity state (13.0 nm) is longer than the reach obtained from the structure of the open conformation (5.3 nm). This suggests that SHP-1 utilizes flexible linkers to achieve a spectrum of open states with a longer reach.

The membrane activity of SHP-1 can be regulated not only by allosteric activation but by the molecular reach, which determines both concentration and coverage (3,24). We found that tethering increases the concentration of SHP-1 from ∼1 *μ*M in solution (cytosol) to over ∼1000 *μ*M when tethered (membrane), but importantly, clustering is necessary for the majority of substrates to experience this high local concentration. Interestingly, this 1000-fold increase is much larger than the twofold increase in the catalytic rate by allosteric activation when the SH2 domain of SHP-1 is engaged.

Although previous reports have demonstrated allosteric activation of SHP-1 using singly phosphorylated peptides engaging a single SH2 domain (9,11,16), recent reports have suggested that allosteric activation of SHP-2 requires simultaneous binding of both SH2 domains on the same (18) or across different PD-1 peptides (17). Although SHP-1 in our assay could in principle bind across two PD-1 peptides, the observed kinetics were characteristic of single SH2 domain binding and not high-affinity tandem SH2 binding, as, for example, observed for ZAP-70 and Syk in SPR (35,36). Moreover, using PD-1 peptides with both ITIM and ITSM phosphorylated produced SPR traces similar to those with only the ITSM phosphorylated (data not shown). Therefore, SHP-1 and SHP-2 may exhibit differences in their allosteric mechanisms.

There is evidence in T cells that SHP-1 and SHP-2 may function through different inhibitory receptors, with PD-1 more readily utilizing SHP-2 compared to SHP-1 (6,7,37). We found that SHP-1 bound to PD-1 with an affinity typical of SH2 domains but that binding was rapidly abolished by autoinhibition in *trans*, whereby SHP-1 dephosphorylated other nearby PD-1 molecules. Although this autoinhibition process was also observed for SHP-2, it took place on the minute timescale and therefore appears to be less efficient than for SHP-1 (5). This may suggest that the interaction of SHP-1 with PD-1 may be important to limit, rather than promote, the activity of PD-1.

Using mathematical modeling, we found that the molecular reach of SHP-1 tethered to inhibitory receptors means that it would only be able to reach 15% of substrates but that coclustering at 10-fold higher density can increase coverage to 90%. Indeed, microscopy experiments have found that inhibitory receptors that can recruit SHP-1 cocluster with their substrates (5,37,38), although the precise density is presently unknown. This result is based on the assumption that inhibitory receptors and their substrates have limited mobility within clusters. We have previously used simulations to show that increasing molecular reach can increase or decrease inhibitory receptor potency when diffusion is slow or fast, respectively (24). Although it is reasonable to expect that the diffusion coefficient of inhibitory receptors would be reduced when they bind their ligands and cluster, direct measurements have yet to be performed. Another mechanism that can potentially control molecular reach within cells is the dynamic and regulated association of the cytoplasmic tails of immune receptors with the membrane (39, 40, 41, 42), which may allow receptor tails to adopt more extended conformations.

The cellular environment is crowded (43,44) and rheologically more complex (45,46) than the fluid environment of our assay. Crowding can effectively change the biophysical parameters that we have reported, including the molecular reach, in a manner that likely depends on the density, size, and shape of the crowding molecules (43,47,48). Although the fluid phase in our assay is dilute, we have noted that it is possible for high concentrations of SHP-1 to accumulate on the surface. This can potentially lead to crowding, explaining why the binding affinity appears to decrease as the SHP-1 concentration increases (Fig. 2 *D*). Studying tethered reactions in the presence of crowding agents in our assay will require overcoming two challenges: extension of the model and simulation to explicitly account for crowding and careful characterization of the relevant in vivo crowding parameters that are to be replicated. Ultimately, these in vitro experiments would benefit from direct in vivo measurements of reach, in which discrepancies between measurements can shed light on both passive and active mechanisms that may be acting in vivo.

The experimental assay and subsequent mathematical analysis we have used can readily be implemented in SPR. An important assumption of the mathematical analysis is that the peptides are randomly distributed. However, given that SPR is based on a flow chamber, it is conceivable that more peptide is deposited near the injection inlet. To reduce this bias, immobilization takes place using a fast flow rate so that a similar peptide concentration is experienced by the entire flow cell. In the future, a complementary method can be used whereby peptides are immobilized at defined distances using DNA origami platforms that themselves are immobilized in SPR. This has recently been used to study antibody-antigen interactions in SPR (49).

It is increasingly clear that cellular signaling relies on tethered reactions (3,19,50,51), and studies have shown how tethering can increase the rate of these intramolecular reactions (20,21). A feature of tethered reactions by immune receptors and many other membrane-confined reactions is that they are intermolecular. This work has highlighted that at typical receptor densities, the short molecular reach of the reaction means that other processes, such as coclustering, are required for efficient signaling, and moreover, small nanometer changes in molecular reach can have large changes on receptor potency. This suggests the possibility of modulating receptor activity by molecular reach inhibitors that can target unstructured receptor tails or flexible linkers within enzymes, which can have advantages over the targeting of structured domains (52, 53, 54).

## Supporting material

Document S1. Figs. S1–S6

Document S2. Article plus supporting material
