# Supplementary material for: Determination of the molecular reach of the protein tyrosine phosphatase SHP-1
Source: Biophys J. 2021 Mar 27;120(10):2054–66. doi: 10.1016/j.bpj.2021.03.019 (PMC8204385; doi:10.1016/j.bpj.2021.03.019)
Supplement: Document S1. Figs. S1–S6 [file mmc1.pdf]

Biophysical Journal, Volume *120*

Supplemental information

Determination of the molecular reach of the protein tyrosine phosphatase SHP-1

Lara Clemens, Mikhail Kutuzov, Kristina Viktoria Bayer, Jesse Goyette, Jun Allard, and Omer Dushek

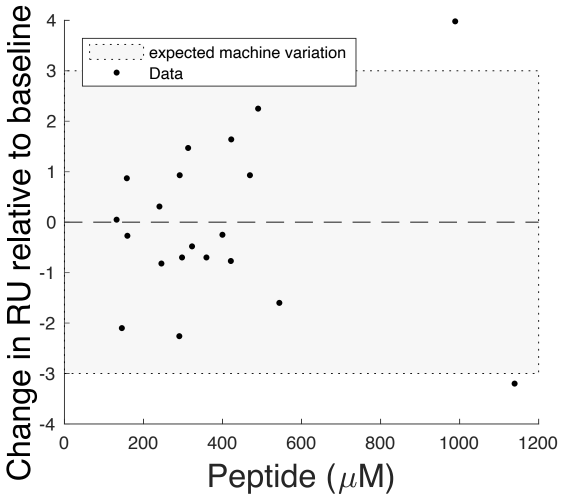

Figure S1: **Change in baseline before and after phosphatase injection is not correlated with phosphorylated peptide concentration.** The observed reduction in baseline RU after SHP-1 injection (see Fig. 2A,B) could be a result of the loss of phosphate mass from the chip surface. To investigate this possibility, alkaline phosphatase was injected over a surface immobilised with the indicated concentration of phosphorylated PEG28-PD-1 peptide for 300 seconds and the difference in baseline RU before and after injection was calculated. However, no correlation was observed with peptide concentration. The small $\sim$3 RU deviation is consistent with machine drift which is reported to be in the range of $\sim$1 RU per 100 seconds (gray area for our 300 second injection).

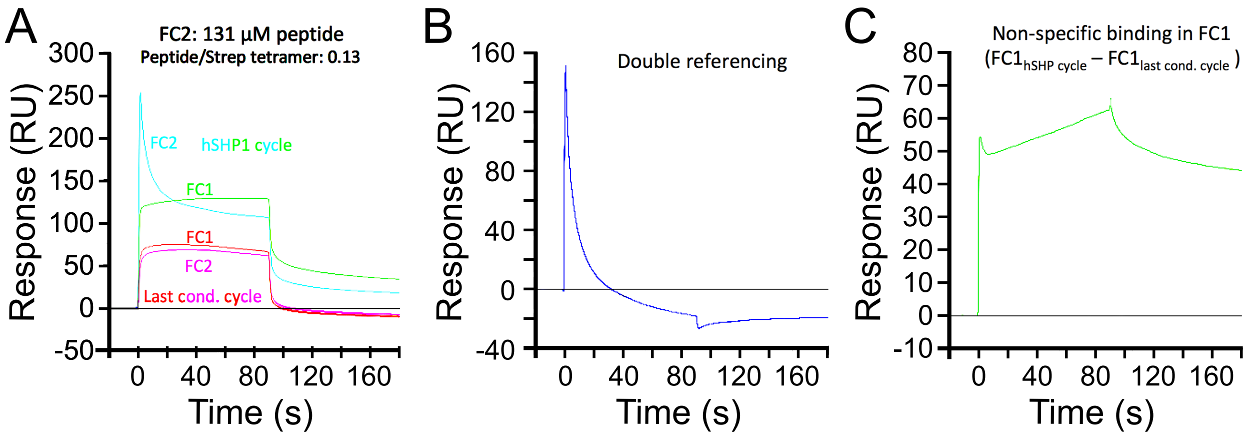

Figure S2: **Change in baseline before and after SHP-1 injection can be explained by non-specific SHP-1 binding.** (A) SPR traces of buffer injection (conditioning cycle) over flow cells 1 (red) and 2 (pink) followed by SHP-1 injection over flow cell 1 (green) and 2 (cyan). Flow cell 1 is a blank control whereas PEG28-PD-1 is immobilised in flow cell 2. All experiments were at at 37°C. Non-specific binding is apparent after SHP-1 injection because baseline remains above zero. (B) Double-referenced SPR trace for PEG28-PD-1 highlights a negative baseline after SHP-1 injection. This result suggests that non-specific binding in the control and experimental flow cells is not exactly matched. (C) Subtraction of the buffer injection from the SHP-1 injection in the control flow cell demonstrates that non-specific binding of SHP-1 can be approximated to be linear in time.

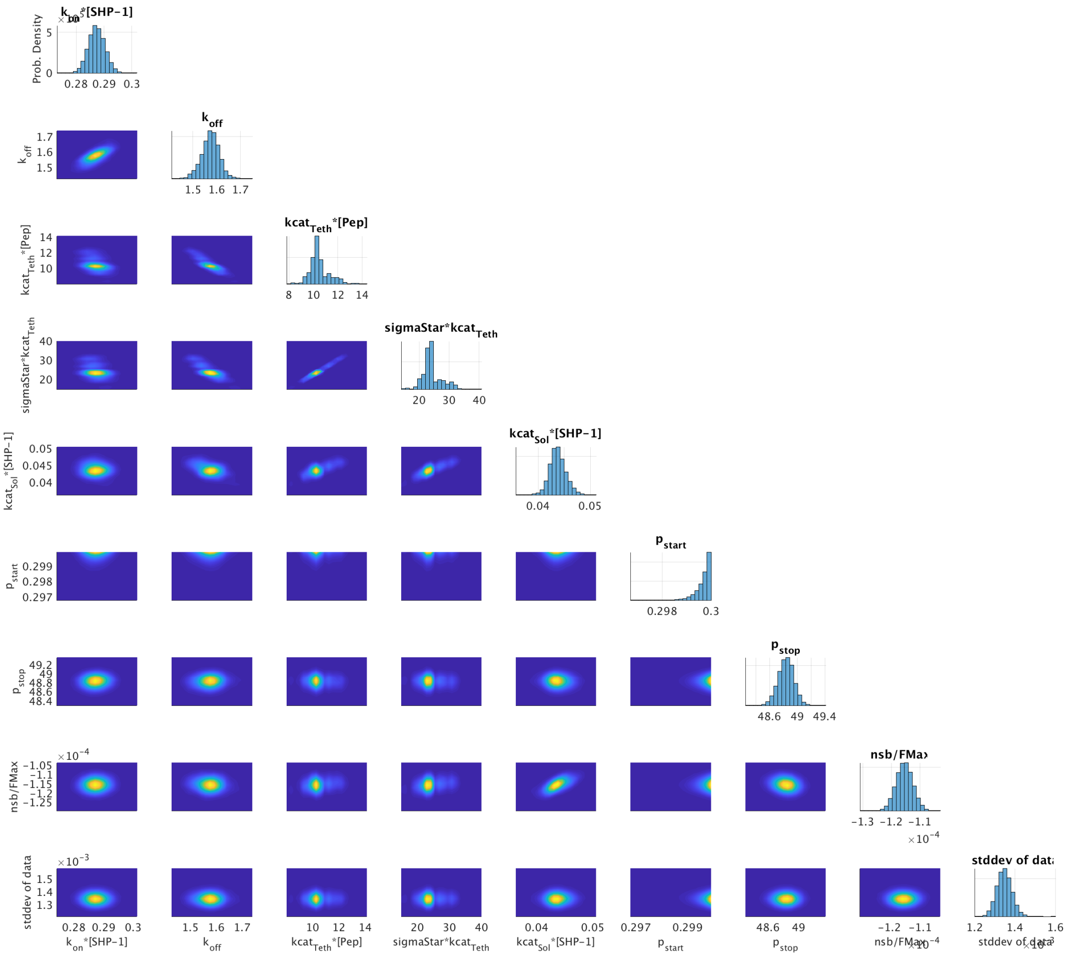

Figure S3: **Posterior distributions of individual fitted parameters.** Using Markov Chain Monte Carlo to fit the MPDPDE model to data obtained at 1 μM SHP-1 and 125 μM PEG28. The compact posterior distributions, in which most of the probability mass is concentrated in a single peak, provides evidence that parameter estimates are well-identified.

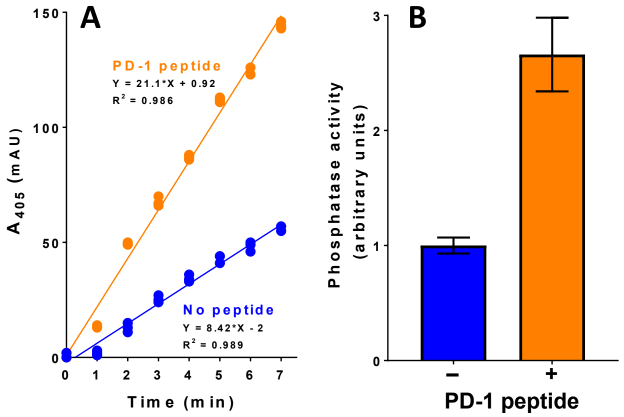

Figure S4: **Solution-based assay for SHP-1 catalytic activity confirms a modest allosteric activation by singly phosphorylated PD-1 peptide.** Human SHP-1 (0.1 μM) was incubated at 37°C with 10 mM pNPP in the absence (blue) or presence (orange) of 60 μM PEG0-PD-1 peptide. (A) Time course of pNPP dephosphorylation measured by absorbance at 405 nm at the indicated time points. Data points are technical replicates (n = 3). (B) The catalytic activity is determined by the fold-change in the slope of the time course data with a mean of 2.7. Error bars represent minimum and maximum from independent experiments (n=2).

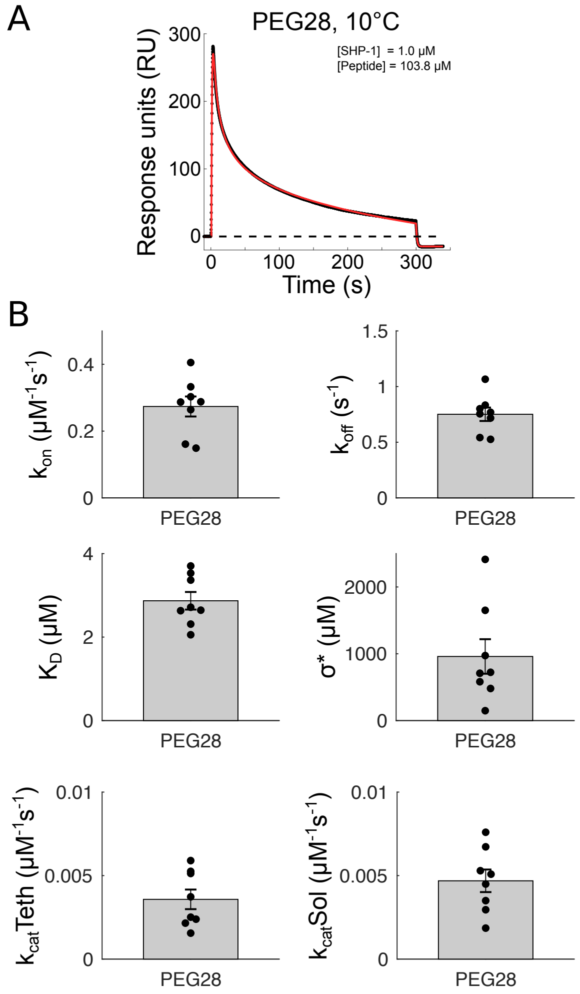

Figure S5: **SHP-1 injection over PEG28-PD-1 at 10°C.** (A) Representative SPR trace (black dots) and extended MPDPDE model fit (red line). (B) Averages and SEMs (gray with black error bars). Individual data points plotted as black dots.

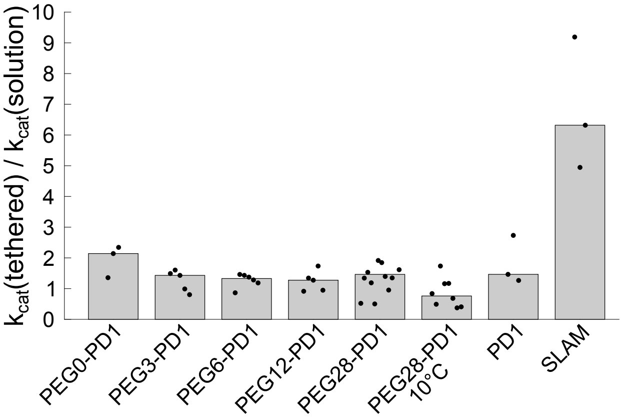

Figure S6: **Summary of k_{cat}(tethered)/k_{cat}(solution) for all experimental conditions.** Ratio of tethered to solution catalysis (k_{cat}(tethered)/k_{cat}(solution)) determined by SPR for the indicated conditions (all experiments at 37°C except where indicated). The median (gray bars) reveals only modest ∼1-2-fold allosteric activation for PD-1 across all conditions but a larger 6.2-fold activation for SLAM, which is discussed in the main text.
